# Advancement and independent validation of a deep learning-based tool for automated scoring of nail psoriasis severity using the modified nail psoriasis severity index

**DOI:** 10.3389/fmed.2025.1574413

**Published:** 2025-04-02

**Authors:** Stephan Kemenes, Liu Chang, Maja Schlereth, Rita Noversa de Sousa, Ioanna Minopoulou, Pauline Fenzl, Giulia Corte, Melek Yalcin Mutlu, Michael Wolfgang Höner, Ioannis Sagonas, Birte Coppers, Anna-Maria Liphardt, David Simon, Arnd Kleyer, Lukas Folle, Michael Sticherling, Georg Schett, Andreas Maier, Filippo Fagni

**Affiliations:** ^1^Department of Dermatology, Friedrich-Alexander-University Erlangen-Nürnberg (FAU) and Universitätsklinikum Erlangen, Erlangen, Germany; ^2^Deutsches Zentrum Immuntherapie (DZI), Friedrich-Alexander-University Erlangen-Nürnberg (FAU) and Universitätsklinikum Erlangen, Erlangen, Germany; ^3^Pattern Recognition Lab, Department of Computer Science, Friedrich-Alexander-Universität Erlangen-Nürnberg, Erlangen, Germany; ^4^Department Artificial Intelligence in Biomedical Engineering, Friedrich-Alexander-Universität Erlangen-Nürnberg, Erlangen, Germany; ^5^Department of Internal Medicine 3 – Rheumatology and Immunology, Friedrich-Alexander-University Erlangen-Nürnberg (FAU) and Universitätsklinikum Erlangen, Erlangen, Germany; ^6^Department of Rheumatology and Clinical Immunology, Charité – Universitätsmedizin Berlin, Berlin, Germany

**Keywords:** psoriasis, psoriatic arthritis, nail disease, NAPSI, MNAPSI, artificial intelligence, machine learning, outcome measures

## Abstract

**Objective:**

To improve and validate a convolutional neural network (CNN)-based model for the automated scoring of nail psoriasis severity using the modified Nail Psoriasis Severity Index (mNAPSI) with adequate accuracy across all severity classes and without dependency on standardized conditions.

**Methods:**

Patients with psoriasis (PsO), psoriatic arthritis (PsA), and non-psoriatic controls including healthy individuals and patients with rheumatoid arthritis were included for training, while validation utilized an independent cohort of psoriatic patients. Nail photographs were pre-processed and segmented and mNAPSI scores were annotated by five expert readers. A CNN based on Bidirectional Encoder representation from Image Transformers (BEiT) architecture and pre-trained on ImageNet-22k was fine-tuned for mNAPSI classification. Model performance was compared with human annotations by using area under the receiver operating characteristic curve (AUROC) and other metrics. A reader study was performed to assess inter-rater variability.

**Results:**

In total, 460 patients providing 4,400 nail photographs were included in the training dataset. The independent validation dataset included 118 further patients who provided 929 nail photographs. The CNN demonstrated high classification performance on the training dataset, achieving mean (SD) AUROC of 86% ± 7% across mNAPSI classes. Performance remained robust on the independent validation dataset, with a mean AUROC of 80% ± 9%, despite variability in imaging conditions. Compared with human annotation, the CNN achieved a Pearson correlation of 0.94 on a patient-level, which remained consistent in the validation dataset.

**Conclusion:**

We developed and validated a CNN that enables the automated, objective scoring of nail psoriasis severity based on mNAPSI with high reliability and without need of image standardization. This approach has potential clinical utility for enabling a standardized time-efficient assessment of nail involvement in the psoriatic disease and possibly as a self-reporting tool.

## Introduction

Psoriasis is a chronic inflammatory disease with highly heterogeneous clinical manifestations predominantly affecting the skin and nails in case of cutaneous psoriasis (PsO), as well as joints, entheses and tendons when Psoriatic Arthritis (PsA) is present (1, 2). Nail involvement is present in approximately 50% of PsO patients (3) and bears substantial prognostic significance. Particulary, its presence has been shown to correlate with disease severity, impaired quality of life, and with the risk of progression to PsA (4, 5). For these reasons, clinical indices measuring the presence and severity of nail psoriasis are commonly used as endpoints in drug trials for PsO and PSA. Thus, an early and accurate assessment of nail psoriasis is of great importance to improve patient management and help guide treatment decisions. Objectively quantifying nail psoriasis requires an assessment of various features of nail disease, including pitting, onycholysis, subungual hyperkeratosis, and nail bed discoloration (3). To do so, comprehensive clinical scores such as the Nail Psoriasis Severity Index (NAPSI) and the modified NAPSI (mNAPSI) have been developed (6, 7). The NAPSI score is the gold standard for assessing nail psoriasis severity by evaluating nail matrix (i.e.: pitting, leukonychia, red spots in the lunula, crumbling) and nail bed changes (i.e.: oil-drop discoloration, onycholysis, hyperkeratosis, splinter hemorrhages). To calculate NAPSI, nails are divided into four quadrants and for each quadrant 1 point can be assigned for each matrix and nail bed changes, resulting in a maximum of 8 points per nail, totaling a range of 0–80 points for 10 nails (6). The mNAPSI is a simplified version of the NAPSI score in which pitting, crumbling, and onycholysis are scored on a semiquantitative 0–3 scale depending on the severity or percentage of nail involved (i.e.: 0 = none, 1 = mild, 2 = moderate, 3 = severe), while splinter hemorrhages, leukonychia, red spots in the lunula, and hyperkeratosis are scored in a binary manner (i.e.: 0 = absent; 1 = present), resulting in a score of up to 13 per nail with a range of 0–130 for 10 nails (7) (Supplementary Figure 1). These scores provide a detailed assessment of all fingernails and are used in clinical trials to assess the effects of treatments and other interventions (8, 9). However, performing such detailed assessment is highly time consuming, which has substantially limited their application outside of clinical trials so far.

In recent years, an increasing number of artificial intelligence (AI) tools have been developed to assist clinical decision making by improving time- and cost-efficiency of various tasks such as the interpretation of imaging and risk stratifications. The first AI-based model for the automatic scoring of NAPSI was developed by Hsieh et al. involved developing an AI system based on nails from 45 patients, using single-nail photos taken in a reflection-free box (10). Similarly, a later model by Paik et al. was based on 7,054 nails and utilized a deep learning algorithm to evaluate NAPSI (11). However, AI models so far have relied on high degrees of standardization for image acquisition and were based on the annotations of a single reader.

In previous work, we addressed some of these limitations by incorporating a multi-reader approach and using mNAPSI (12). This work, which served as the precursor to our current model, improved scoring accuracy but the need for highly standardized, reflection-free photos and the lack of an external validation remained major limitations, reducing its applicability in everyday use. Furthermore, the relatively low number of severely affected nails limited its accuracy on higher mNAPSI classes. Building on this, in this study we aimed to train and validate a new convolutional neural network (CNN)-based model with the objective of improving accuracy throughout all classes of NAPSI severity as well as of decreasing the need of picture standardization. To do so, we employed a larger dataset of hand photographs including more patients with severe disease and acquired without standardized conditions. Additionally, we validated our findings on an independent external cohort.

## Methods

### Patient selection

All participants to this study were recruited at outpatient clinics of the Department of Internal Medicine 3, Rheumatology and the Department of Dermatology of the University Hospital of Erlangen between January 2022 and December 2023. Only adult participants were included. To be suitable for inclusion in the study (i.e., for both training and validation cohort), patients with PsO had to have a biopsy-proven or dermatologist-confirmed diagnosis of psoriasis. PsA patients had to fulfill the ClASsification for Psoriatic ARthritis (CASPAR) criteria for PsA (13). Patients with rheumatoid arthritis (RA) were included as controls and had to fulfill the ACR/EULAR 2010 classification criteria (14). Healthy controls (HC) were recruited from the local community. Individuals suffering from health conditions that affect the nails other than PsO and PsA (e.g., untreated hypothyroidism, traumatic nail dystrophy) were excluded from participation. The cohort used for the training of the model was recruited at the outpatient clinic of the Department of Rheumatology and Immunology and included patients with PsO, PsA, RA, as well as HC. This cohort comprised part of the participants included in our previous study (12). The validation cohort only included patients with PsO with or without nail psoriasis recruited at the outpatient clinic of the dermatology department. All participants signed a written informed consent for participation. This study was conducted according to the principles of the Declaration of Helsinki and was approved by the Ethics Committee of the University Hospital of Erlangen (#21-422-B). All participants provided written informed consent for the scientific use and publication of their photographs.

### Photograph acquisition, segmentation, and processing

Hand photographs for the dataset were collected in a three-stage process. Images from the first two stages were used for training, while images from the third stage were obtained by an external cohort at our dermatology department and were used for validation. In the first stage photographs were scored by three rheumatologists (FF, PF, AK), in the second stage by a fourth rheumatologist (RNS), and finally the third stage used for validation was annotated by a trained dermatologist (SK). In the first and second stage, all hand photographs were captured using the same iPad Pro (iOS 14, Apple, USA) device under standardized lighting and background using a custom photo box, as described elsewhere (12) (Supplementary Figure 2). In the third stage, photographs were taken without any standardization in terms of background, lighting, distance, angle, and focus using either the iPad Pro or an iPhone device (iOS 14, Apple, USA).

**Figure 1:**
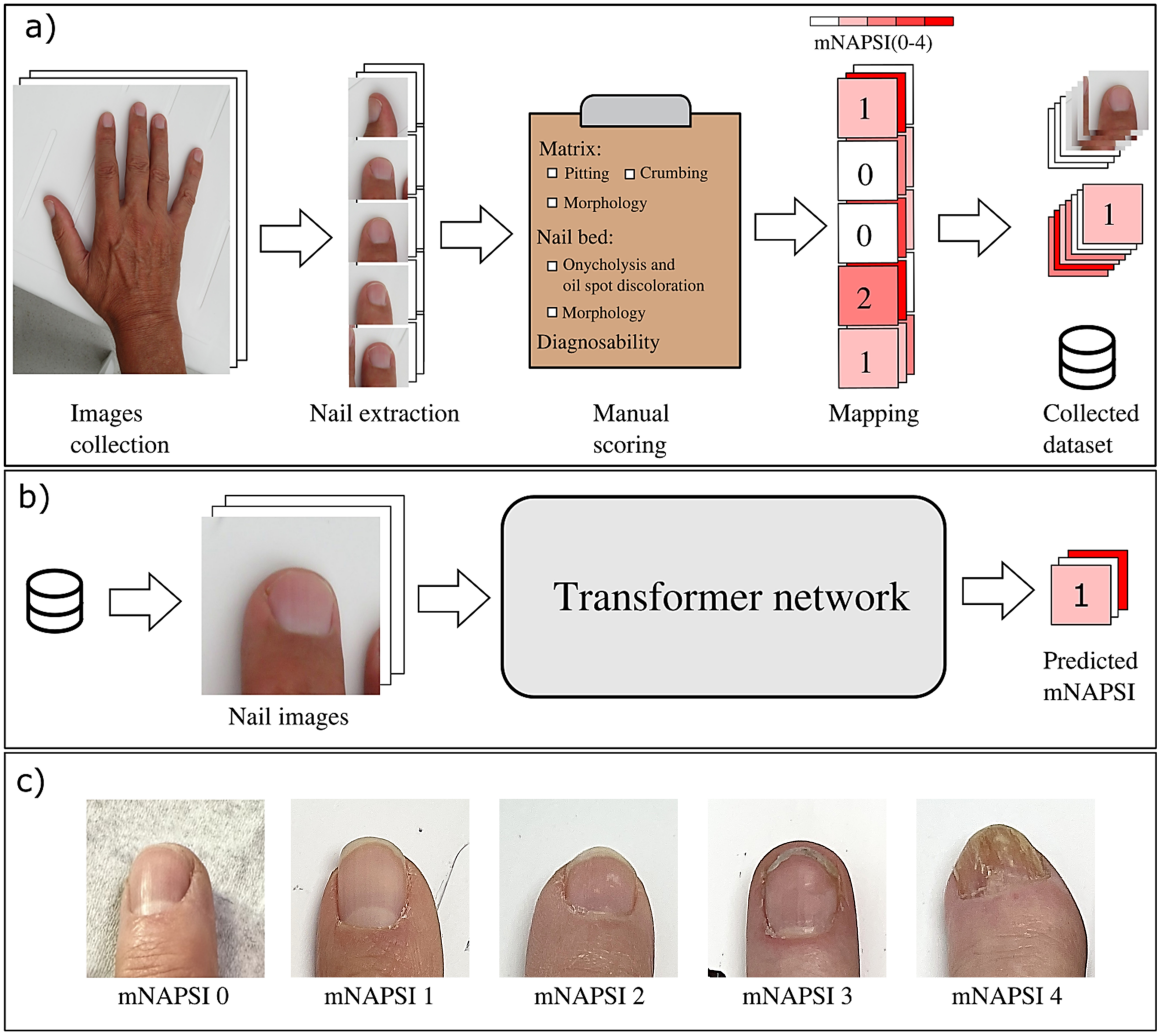
Pipeline of the data collection **(a)** and of the model training process **(b)**. **(c)** Shows visual examples of nail changes throughout the different mNAPSI classes 1–5.

To prepare the nail photos for the training of the neural network, all hand photographs are pre-processed to extract only the nail region of the hand photograph, which is the only one relevant for mNAPSI classification. To do so, we used the MediaPipe framework’s hand key point detection algorithm (15) (Google, USA), which identifies the nails by localizing the key points on each finger and selecting the most distal ones. The orientation of the extracted nail picture was standardized by rotating it based on the angle determine by the most distal and second most distal key point of the finger. After this, images are resized to fit uniform size. This process results in a set of ten normalized nail images per patient, as show in Figure 1. Further details to this process have been described elsewhere (12).

### Clinical evaluation of nail disease with mNAPSI

To evaluate nail psoriasis severity, mNAPSI was used. Accordingly, changes in the nail were scored semi-quantitatively on a 0–3 scale: pitting (0 = 0, 0–10 = 1, 11–49 = 2, 50+ =3), crumbling (0% = 0, 1–25% =1, 26–50% =2, 51–100% = 3), onycholysis (0% = 0, 1–10% = 1, 11–30% = 2, 31–100% = 3). Ultimately, this results in a score ranging from 0 to 9 for each nail, totaling a maximum of 90 for both hands. In addition, the annotators are asked to evaluate the diagnosability of each nail image on a 1–3 scale including “good diagnosability” (=1), “acceptable diagnosability” (=2), and “non diagnosable” (=3).

For our analysis, a total of five physicians trained in performing the mNAPSI score were asked to score each nail independently. Picture of all nails were randomly assorted and presented to the readers in no particular order to minimize the risk of assigning lower or higher scores on a patient-by-patient basis. The readers were blinded to diagnosis, sex, age, and clinical history of all patients. Regarding the external cohort used for validation, nail photographs were presented in an analogous manner to a trained dermatologist who was also blinded to sex, age, and clinical history of the patients but not to diagnosis, as all patients had either PsO or PsA with or without nail involvement.

### Deep learning architecture and model training

An overview of the pipeline of building up the dataset as well as the model training is depicted in Figure 1. Briefly, after the annotation of all mNAPSI scores through the readers, the deep learning model is trained using images from the training dataset to predict the grouped mNAPSI score between 0 and 4. Since the number of nail images with mNAPSI scores higher than 4 are limited compared with those with lower scores, images with a score of 4 or higher were aggregated into the same class (i.e., mNAPSI class 5) to mitigate the imbalance of the dataset. Nail images with diagnosability scored as “non diagnosable” were eliminated. We based our CNN on the transformer-based BEiT (Bidirectional Encoder representation from Image Transformers) (16). The network architecture is the same as the original vision transformer, with image tokenization and patch embedding to elaborate the global information in images. Next, we tested the pre-trained BEiT to carry out the nail classification task. The pre-trained model underwent a combined process of self-supervised learning and training on the large-scale ImageNet-22 k dataset to ensure its generalizability, allowing it to be fine-tuned for new vision tasks. The fine-tuning of the hyper-parameter on a subset of the dataset resulted in the following configurations: Adam optimizer, a learning rate of 9e-6, and a weight decay of 1e-3. Training was terminated when the validation AUROC did not further improve for 100 training epochs. All the models were trained on one Nvidia A100 (40G). To further enhance the model’s performance, five networks were trained on the same dataset with different initialization. The ensemble of the five models was then used to perform the final score prediction.

### Experiments

To enhance the robustness of the neural network against the high variability expected in our dataset, we applied a data augmentation process. This included random flipping along both vertical and horizontal axes, random rotations within ±10 degrees, random translations of up to 10% in each direction, random scaling within ±10%, and random shearing up to 2 degrees.

For model evaluation, the dataset was divided into three stages. The first two stages were split into training and test sets, while the third stage, captured using a different device, was used exclusively for evaluation. To assess the impact of dataset size, we compared a model trained using only the first stage’s training data with a model trained using data from both the first and second stages. Additionally, the generalizability of the model was evaluated by comparing its performance on the test data from the first two stages with its performance on the third stage.

### Reader study

A reader study was conducted to assess the interrater variability among the five expert readers using a subset of 100 nail photographs. Inter-rater reliability for the mNAPSI scores, aggregated at the patient level, was evaluated using Cronbach’s alpha. The correlation between the expert readers’ mNAPSI ratings and the network-based predictions was assessed using the Pearson correlation coefficient (Supplementary Figure 3).

### Statistical methods

Patient characteristics were represented as summary statistics for continuous and categorical data. We utilized Pearson correlation and the least-squares fit analyses to assess correlation between human annotation and predicted mNAPSI. To compare the accuracy of the classification performed by the algorithm against a reference value expected in case of a random classification, we used the macro area under the receiver operating characteristics curve (AUROC). The macro-AUROC measures how well a model distinguishes between positive and negative cases based on various thresholding settings for each class separately, avoiding strong influence from the dominant class. The reference value for the AUROC in case of a random classification is AUOC ≤0.5. The F1 score represents the harmonic mean between precision and recall while the mean average error (MAE) is the average of all absolute errors. Sensitivity, Specificity, Precision, and Accuracy were calculated for the prediction of each mNAPSI class. Lastly, the weighted precision-recall (PR)-AUC was calculated, which summarizes the area under the Precision-Recall curve, i.e., the trade-off between positive predictions and true positive rate depending on the class frequency.

## Results

### Patient characteristics

We included 638 patients providing a total of 5,357 nail images. In the first stage we included 177 patients with 1770 nail images, 283 patients with 2,630 nail images in the second stage, and 118 patients with 929 nail images in the third phase. In the first phase 1,227 nail images were scored good in diagnosability, while 412 as acceptable and 138 were not evaluable due to lacking image quality. In the second phase, 1883 nails are marked as good, 366 as acceptable and 374 as not diagnosable. In the third phase, 391 nails are marked as good, 433 as acceptable and 105 as not diagnosable.

**Figure 2:**
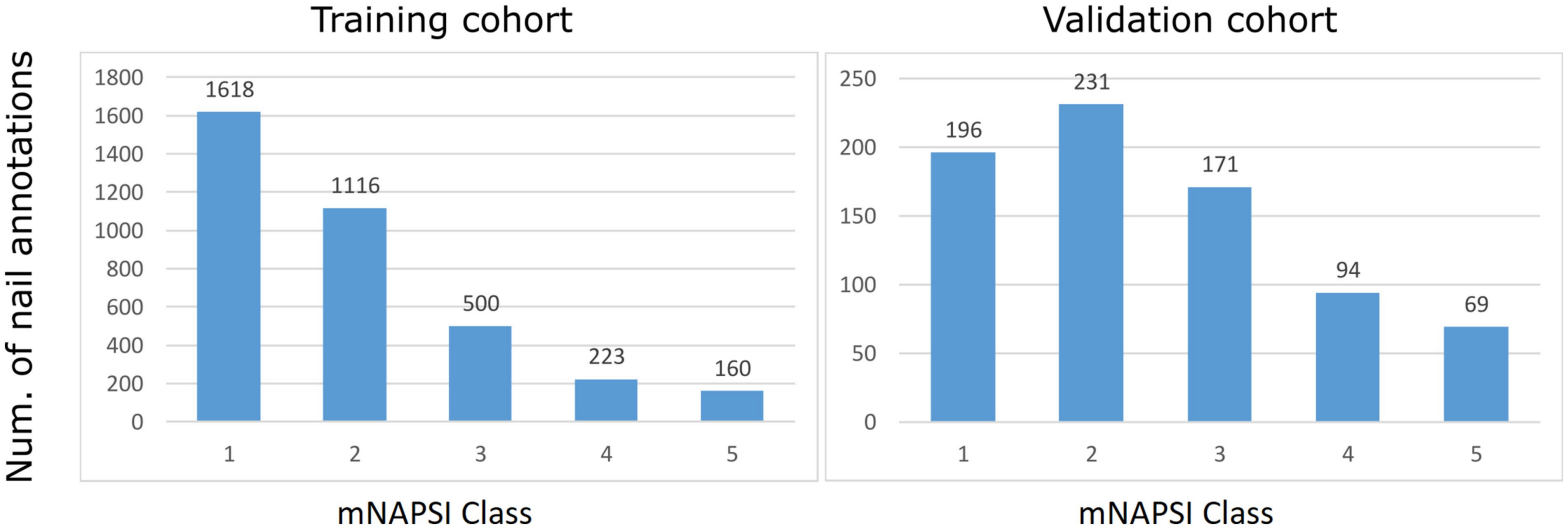
Histograms of the mNAPSI annotation of collected nails.

Patients recruited in the first two stages were included in the training dataset. The training population had a female preponderance (60.0%) and a mean (standard deviation (SD)) age of 53.7 (14.2) years. All patients were of Caucasian ethnicity with low skin phototype (Fitzpatrick I-III). Most patients had PsA (*n* = 155, 35.8%) or cutaneous PsO (*n* = 49, 11.3%) without arthritis. Non-psoriatic controls included RA (*n* = 176, 40.6%) and HC (*n* = 53, 12.2%). The mean (SD) mNAPSI score in the training dataset was 8.3 (6.8). An exact overview of the mNAPSI classes for the training dataset is provided in Figure 2. Patients from the third stage constituted the external validation dataset. Sex distribution and age were comparable to the training dataset, with females slightly overrepresented (55.1%) and a mean age of 50.1 (10.3) years (differences not significant). All patients from the validation cohort had a diagnosis of PsO (*n* = 178, 100%) and the mean (SD) mNAPSI was 10.02 (5.9), which was significantly higher than the training cohort (*p* < 0.001), likely due to the absence of non-psoriatic controls.

### Performance of the algorithm on the training dataset

**Figure 3:**
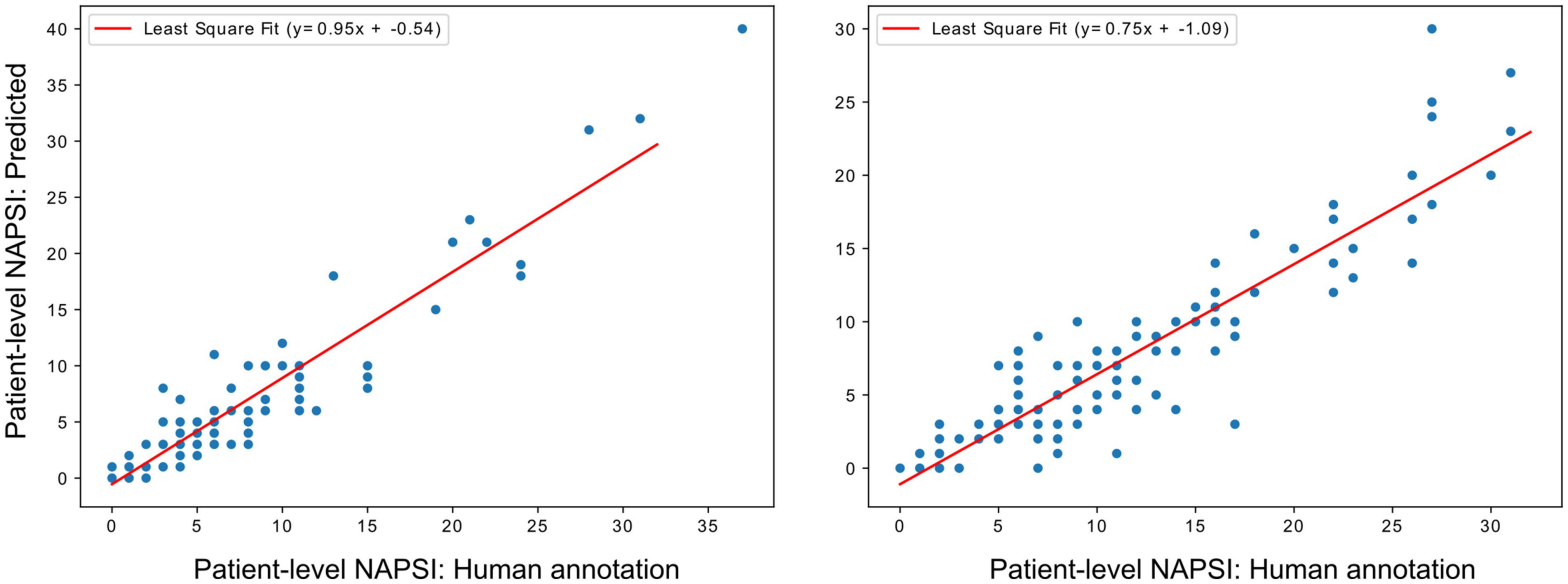
Least-squares fit analyses of human annotation against CNN-predicted mNAPSI at patient-level for training (left panel) and validation datasets (right panel).

The model’s performance on the training dataset is presented in Table 1 and reports metrics for each mNAPSI class as well as the overall average. We observed a high correlation of 0.94 (*p* < 0.001) for mNAPSI scoring between human annotation and CNN, indicating a strong agreement on a single nail level. The least-squares fit analyses resulted in a slope of 0.95 and an intersection of −0.54, indicating that the predictive capability for mNAPSI is very high, with a slight underestimation of lower scores (Figure 3, left panel).

**Table 1:** Demographic and clinical characteristics of the cohorts.

		Training cohort	Validation cohort
Number of patients	*n*	460	118
Sex	Female, *n* (%)	276 (60%)	98 (55.1)
	Male, *n* (%)	184 (40%)	80 (44.9)
Age	Years, mean (SD)	53.7 (14.2)	50.1 (10.3)
Diagnosis	Psoriatic Arthritis, *n* (%)	155 (35.8%)	-
	Psoriasis, *n* (%)	49 (11.3%)	178 (100.0%)
	Rheumatoid Arthritis, *n* (%)	176 (40.6%)	-
	Healthy controls, *n* (%)	53 (12.2%)	-
Nail photographs	*n*	4,400	929
	Good diagnosability, *n* (%)	3,110 (70.7)	391 (42.1)
	Acceptable diagnosability, *n* (%)	778 (17.7)	433 (46.6)
	Non-diagnosable, *n* (%)	512 (16.6)	105 (11.3)
mNAPSI	mean (SD)	8.3 (6.8)	10.2 (5.9)

**Figure 4:**
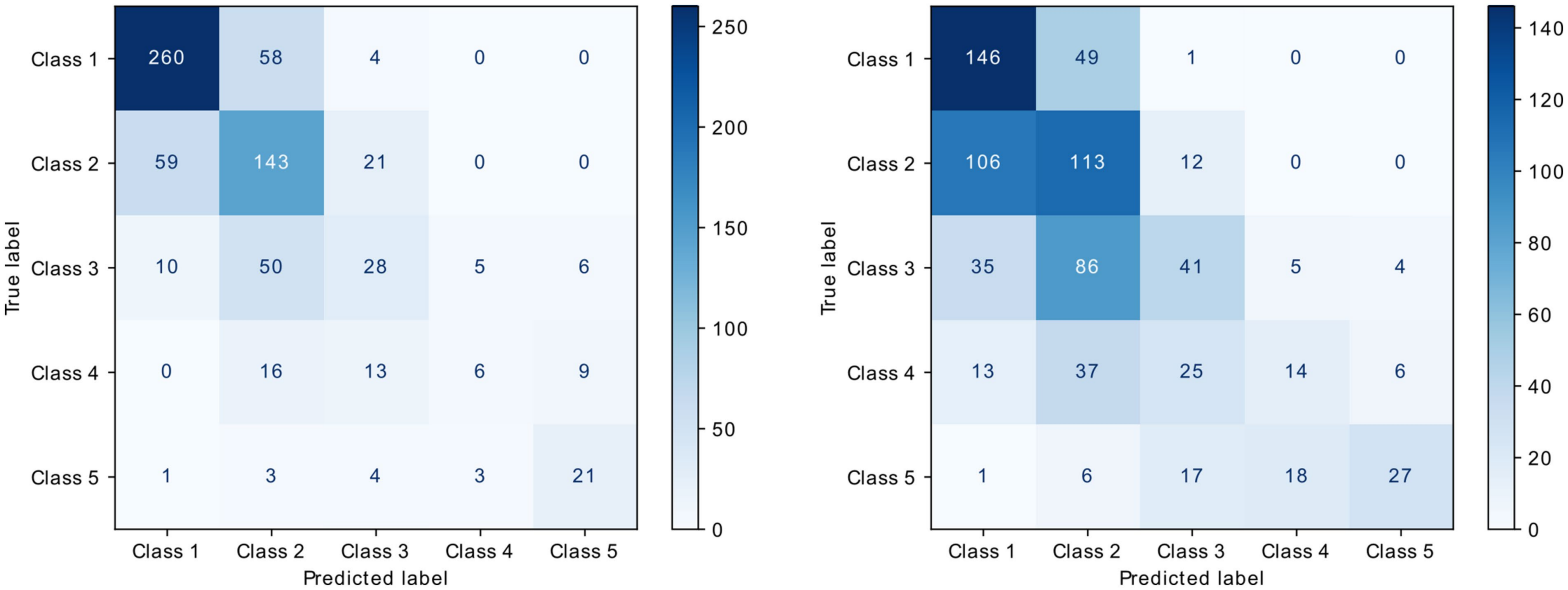
Confusion matrix of the validation dataset (right) and test data from training dataset (left).

**Figure 5:**
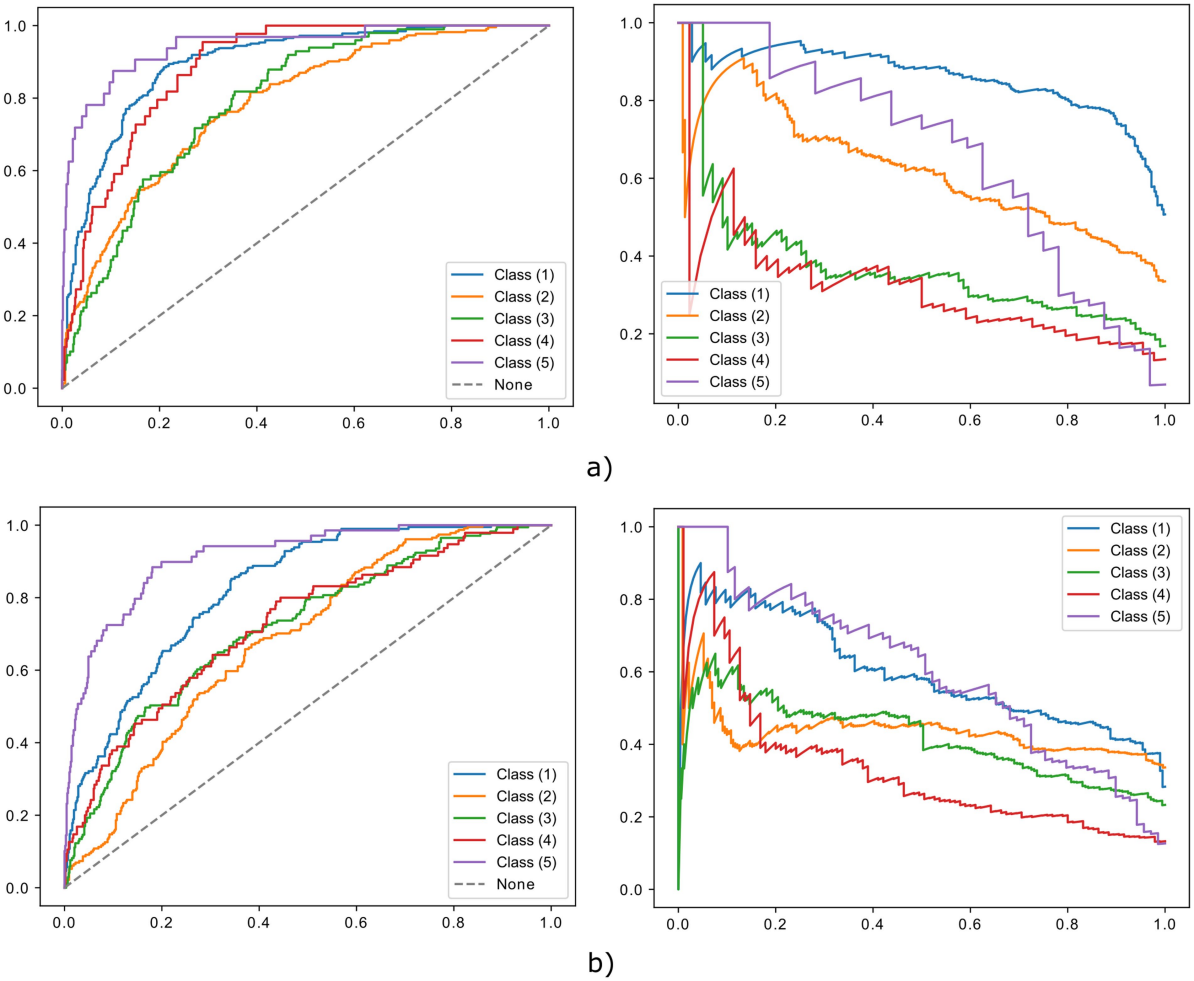
ROC (left) and PRC (right) curves of the training **(a)** and validation **(b)** dataset.

Accordingly, the CNN achieved a high classification performance throughout all classes, with an average (±SD) AUROC of 0.862 (0.236) and average accuracy of 0. 636 (±0.282) throughout classes. The CNN reached the best performance in classifying the lowest and highest mNAPSI scores, for which high AUROC and accuracy were obtained. Good AUROC was also achieved for mNAPSI classes 3 and 4 despite a drop in accuracy. Class 1 mNAPSI was identified with an AUROC of 0.898 and accuracy of 0.807, Class 2 mNAPSI with an AUROC of 0.785 and accuracy of 0.641, Class 3 mNAPSI with an AUROC of 0.794 and an accuracy of 0.283, Class 4 mNAPSI with an AUROC of 0.887 and accuracy of 0.136, and Class 5 mNAPSI with an AUROC of 0.945 and accuracy of 0.656. These performances are further reflected in the ROC and PRC curves (Figure 4A), where the performance of class 1, 4, and 5 stand out. Figure 5 (left panel) shows a confusion matrix of the training dataset providing detailed insight into correct and false predictions. The main weight of the matrix is distributed among the diagonal axis, which corresponds to correctly predicted mNAPSI scores.

### Performance of the algorithm in the independent validation dataset

The model’s performance on the validation dataset is presented in Table 2. Correlation between human annotation and CNN-predicted classification remained high in the validation dataset with *r* = 0.92 (*p* < 0.001). Compared to training, the least-squares fit analysis showed a slightly reduced slope of 0.75 and an intersect of −1.09. This suggests that a good correlation with human annotation is retained, with underestimation of lower mNAPSI scores (Figure 3, left panel).

**Table 2:** Classification performance of the neural network on the training dataset.

	AUROC	MAE	F1	Precision	Sensitivity	Specificity	Accuracy
Class 1	0.898		0.798	0.859	0.807	0.824	0.807
Class 2	0.785		0.580	0.620	0.641	0.744	0.641
Class 3	0.794		0.331	0.375	0.283	0.932	0.283
Class 4	0.887		0.207	0.323	0.136	0.988	0.136
Class 5	0.945		0.618	0.667	0.656	0.978	0.656
Average (macro)	0.862		0.507	0.569	0.505	0.893	0.505
Std	0.070		0.236	0.221	0.282	0.106	0.282
Average (micro)		0.432	0.636	0.569	0.636	0.909	0.636

Class-wise and average metrics showed an expected slight performance drop compared to the training dataset, with an average AUROC of 0.801 and average accuracy of 0.434. The classification performance remained high throughout all classes, with Class 1, 4, and 5 remaining the best classified by AUROC and accuracy also in the validation dataset. Class 1 mNAPSI was identified with an AUROC of 0.820 and accuracy of 0.745, Class 2 mNAPSI with an AUROC of 0.679 and accuracy of 0.497, Class 3 mNAPSI with an AUROC of 0.352 and an accuracy of 0.283, Class 4 mNAPSI with an AUROC of 0.843 and accuracy of 0.250, and Class 5 mNAPSI with an AUROC of 0.923 and accuracy of 0.394. Notably, while the performance on Class 2 slightly declined, the classification of Class 3 remained grossly stable, and the accuracy of class 4 even increased. Figure 3 shows the ROC and PRC curves of the validation dataset in comparison to those of the training dataset in the upper panel. A confusion matrix of the validation dataset is provided in Figure 5 (right panel).

### Impact of training data size on neural network performance

**Figure 6:**
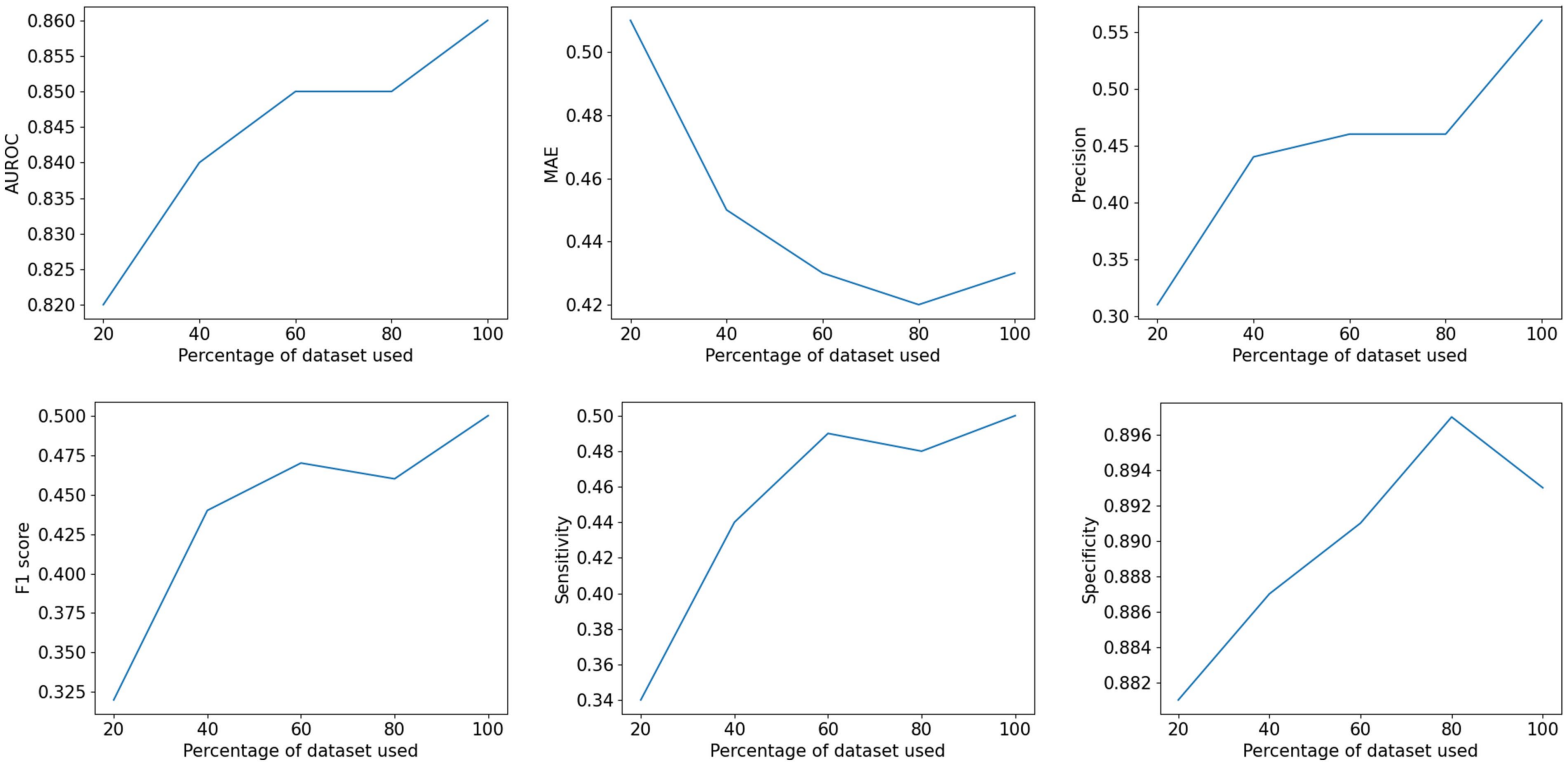
Influence of training set sizes on the performance of the neural network.

A subset analysis was conducted on the image data from the first two phases to evaluate the impact of training data size. Random subsets comprising 20%, 40%, 60%, and 80 of the training data were sampled, and models were trained using the same configuration (Figure 6).

The relationship between classification metrics and training data size is illustrated in Figure 5. AUROC, F1 score, and MAE all improve as the training data size increases. Notably, sensitivity improves by 47% when comparing the 20% subset to the full dataset, while specificity shows a modest improvement of only 1.5%. This indicates that the enhanced classification performance primarily stems from better detection of psoriatic nails. Overall, the subset analysis demonstrates that increasing the training data size enhances the robustness of the classification network (Table 3).

**Table 3:** Classification performance of the neural network on the external validation dataset.

	AUROC	MAE	F1	Precision	Sensitivity	Specificity	Accuracy
Class 1	0.820		0.639	0.670	0.745	0.704	0.745
Class 2	0.679		0.479	0.461	0.497	0.710	0.497
Class 3	0.740		0.352	0.391	0.283	0.922	0.283
Class 4	0.843		0.329	0.373	0.250	0.976	0.250
Class 5	0.923		0.491	0.574	0.394	0.987	0.394
Average (macro)	0.801		0.458	0.494	0.434	0.860	0.434
Std	0.095		0.125	0.126	0.199	0.141	0.199
Average (micro)		0.598	0.513	0.494	0.513	0.878	0.513

### Reader study

The agreement within the five readers was good and achieved a Cronbach’s alpha of 80% (95% CI: 0.71–0.865), which is in line with previous reports (12). The pairwise Pearson correlations between readers showed *r* coefficients ranging from 0.32 and 0.76. All but one were significant with *p* values <0.01. Correlation between reader 3 and 5 was not significant (Supplementary Table 1).

## Discussion

In this study, we successfully trained a CNN-based model to automatically predict and score nail disease severity in psoriatic disease by mNAPSI. Furthermore, we could successfully validate the model on an independent external cohort of nail photographs taken without any standardization in light, angle, and exposure.

The clinical interest in developing AI-based tools to automatically and objectively determine disease activity parameters in psoriasis, PsA, and other forms of arthritis is rising, but the clinical applicability of these approaches has remained limited so far. CNNs have already been successfully developed and validated to classify different forms of arthritis on high-resolution computed tomography (17) and MRI (18), as well as to score disease activity based on ultrasound (19, 20), X-rays (21), and MRI (18, 22). AI tools addressed at other clinical disease activity measures of PsA such as skin and nail disease are still underresearched, but there have been promising developments so far. For instance, Horikawa at al trained a model that slightly outperformed dermatologists in rating NAPSI (23), indicating that a standardization of nail disease assessment *via* AI-based tools is feasible.

In our photography-based model, the CNN reached high AUROC in all mNAPSI classes and correlated strongly with human annotation. Compared to our previous work, we were able to substantially improve the performance of the classification model throughout all mNAPSI classes. Notably, the validation of the CNN on an independent cohort of hand photographs remained consistent with the ones observed during training despite the lack of standardization of the photographic conditions. The overall class performance of the CNN only slightly dropped during validation and showed some improvement in the accuracy of the intermediate classes.

Our experiments have some limitations. First, the training and validation datasets were derived from two centers at the same university. Second, intermediate mNAPSI severity classes (i.e., mNAPSI classes 2, 3, and 4) were more often misclassified compared to the extremes of the severity spectrum. This may be due to the higher abundance of class 1 nails, which resulted in better performance for this category, and the aggregation of multiple severity levels into class 5, reducing the likelihood of misclassification for high mNAPSI scores. Nonetheless, other sources of bias such as the influence of reader bias can be considered neglectable in our case, as the results of the reader study showed overall high agreement between all individual human annotators. Additionally, the CNN was not tested on other nail pathologies and may yield false positives if conditions other than psoriasis are causing the nail changes. As such, this model in its current version should only be used for psoriatic patients. Finally, all patients were of Caucasian ethnicity, which might limit the performance of the algorithm in patients with darker skin tones.

To improve the accuracy and applicability of similar AI-based tools on real-life cohorts, CNNs will need to be trained to discern between nail conditions (e.g., onychomycosis, nail dystrophy, etc.). In general, training the CNN on larger, more diverse datasets of unstandardized photographs from multiple centers taken with different devices will be indispensable for improving the generalizability of the algorithm. Lastly, longitudinal validation in prospective cohorts is necessary to test and improve the model’s sensitivity to change, such as detecting nail changes in the same patient after starting therapy.

In conclusion, our validated CNN enables an automated and unbiased scoring of nail disease severity in patients with psoriasis and PsA. If successfully developed, our approach can be translated into clinical practice and has the potential to be implemented in app-based self-reporting tools. To facilitate the diffusion of our CNN, we will make its architecture available on request.

## Data Availability

The raw data supporting the conclusions of this article will be made available by the authors, without undue reservation.
