# Corrigendum: Advancement and independent validation of a deep learning-based tool for automated scoring of nail psoriasis severity using the modified nail psoriasis severity index

**DOI:** 10.3389/fmed.2025.1617441

**Published:** 2025-06-05

**Authors:** Stephan Kemenes, Liu Chang, Maja Schlereth, Rita Noversa de Sousa, Ioanna Minopoulou, Pauline Fenzl, Giulia Corte, Melek Yalcin Mutlu, Michael Wolfgang Höner, Ioannis Sagonas, Birte Coppers, Anna-Maria Liphardt, David Simon, Arnd Kleyer, Lukas Folle, Michael Sticherling, Georg Schett, Andreas Maier, Filippo Fagni

**Affiliations:** ^1^Department of Dermatology, Friedrich-Alexander-University Erlangen-Nürnberg (FAU) and Universitätsklinikum Erlangen, Erlangen, Germany; ^2^Deutsches Zentrum Immuntherapie (DZI), Friedrich-Alexander-University Erlangen-Nürnberg (FAU) and Universitätsklinikum Erlangen, Erlangen, Germany; ^3^Pattern Recognition Lab, Department of Computer Science, Friedrich-Alexander-Universität Erlangen-Nürnberg, Erlangen, Germany; ^4^Department Artificial Intelligence in Biomedical Engineering, Friedrich-Alexander-Universität Erlangen-Nürnberg, Erlangen, Germany; ^5^Department of Internal Medicine 3 – Rheumatology and Immunology, Friedrich-Alexander-University Erlangen-Nürnberg (FAU) and Universitätsklinikum Erlangen, Erlangen, Germany; ^6^Department of Rheumatology and Clinical Immunology, Charité – Universitätsmedizin Berlin, Berlin, Germany

**Keywords:** psoriasis, psoriatic arthritis, nail disease, NAPSI, MNAPSI, artificial intelligence, machine learning, outcome measures

In the published article, an author name was incorrectly written as Anna-Maria Liphart. The correct spelling is Anna-Maria Liphardt.

The authors apologize for this error and state that this does not change the scientific conclusions of the article in any way. The original article has been updated.

