# Supplementary figures and images for: Advancement and independent validation of a deep learning-based tool for automated scoring of nail psoriasis severity using the modified nail psoriasis severity index
Source: Front Med (Lausanne). 2025 Apr 2;12:1574413. doi: 10.3389/fmed.2025.1574413 (PMC12000154; doi:10.3389/fmed.2025.1574413)

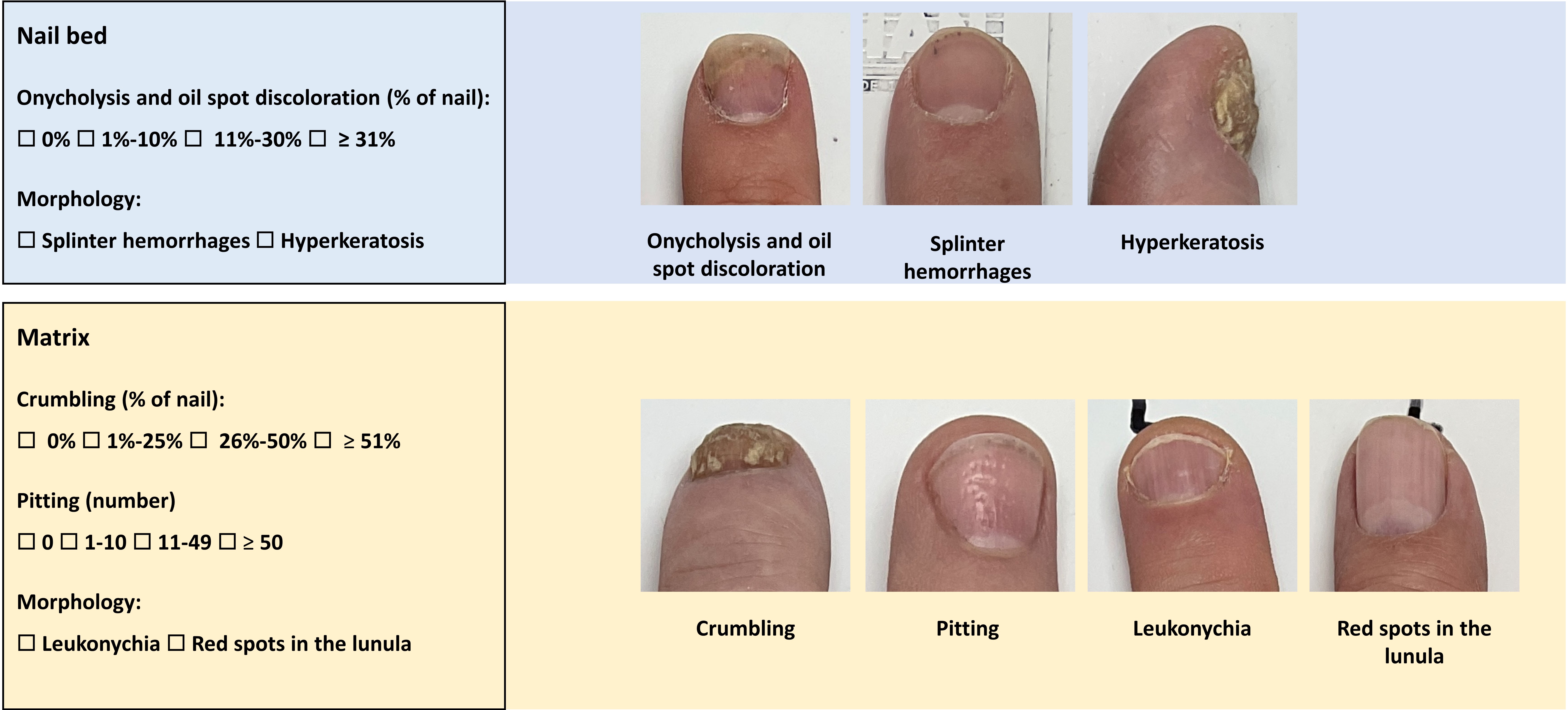

Supplement: Supplementary file 1 [file Image_1.JPEG]

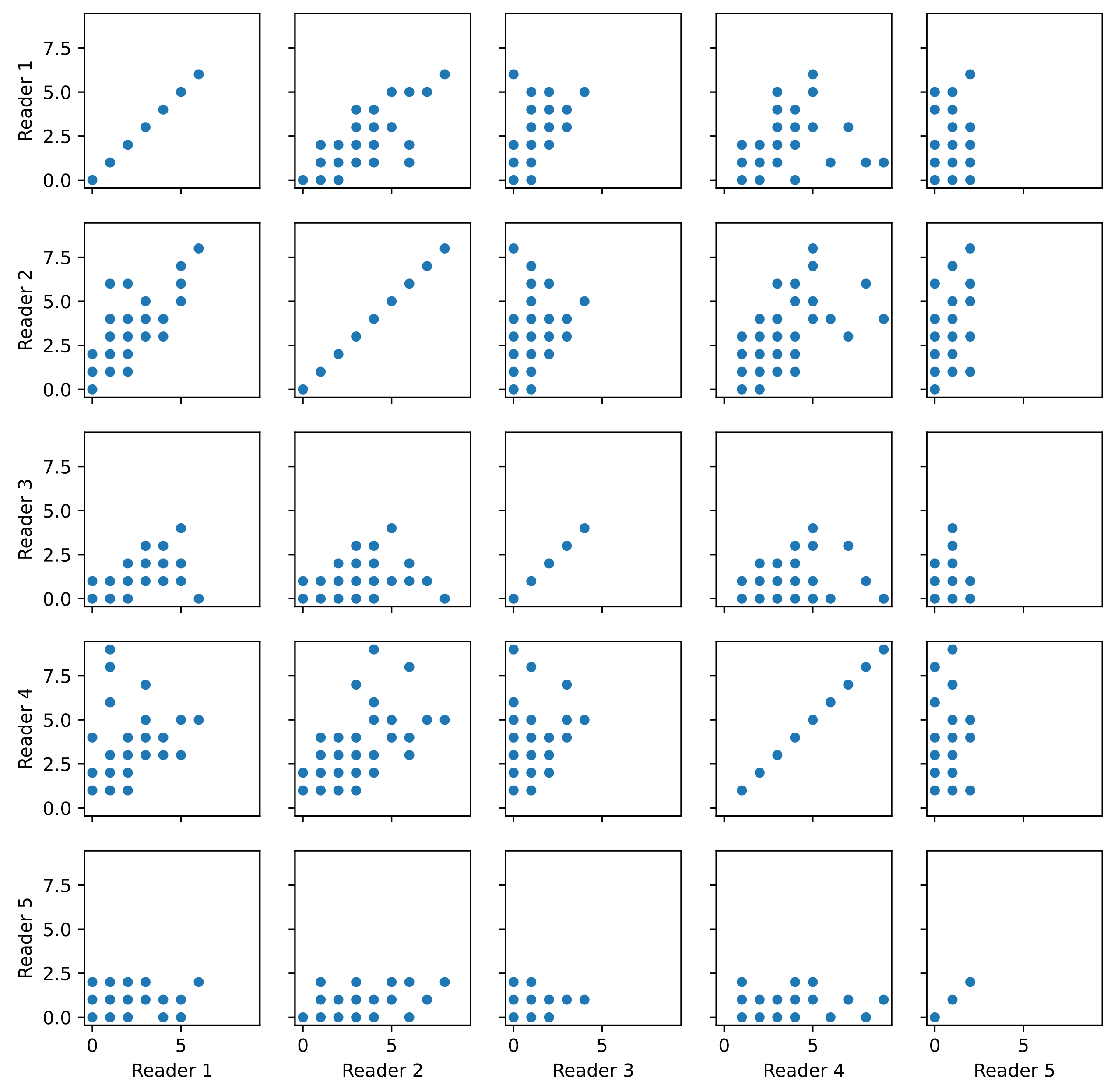

Supplement: Supplementary file 3 [file Image_3.JPEG]
