# Supplementary material for: Advancement and independent validation of a deep learning-based tool for automated scoring of nail psoriasis severity using the modified nail psoriasis severity index
Source: Front Med (Lausanne). 2025 Apr 2;12:1574413. doi: 10.3389/fmed.2025.1574413 (PMC12000154; doi:10.3389/fmed.2025.1574413)
Supplement: Supplementary file 4 [file Data_Sheet_1.docx]

Supplementary material to:

**Development and Independent Validation of a Deep Learning-based tool for Automated Scoring of Nail Psoriasis Severity Using the Modified Nail Psoriasis Severity Index**

Stephan Kemenes^1,2^, Liu Chang^3^, Maja Schlereth^4^, Rita Noversa de Sousa^2,5^, Ioanna Minopoulou^6^, Pauline Fenzl^2,5^, Giulia Corte^2,5^, Melek Yalcin Mutlu^2,5^, Michael Wolfgang Höner^1,2^, Ioannis Sagonas^1,2^, Birte Coppers^2,5^, Anna-Maria Liphart^2,5^, David Simon^6^, Arnd Kleyer^6^, Lukas Folle^3^, Michael Sticherling^1,2^, Georg Schett^2,5^, Andreas Maier^3^, Filippo Fagni^2,5^

^1^Department of Dermatology, Friedrich-Alexander-University Erlangen-Nürnberg (FAU) and Universitätsklinikum Erlangen, Erlangen, Germany

^2^Deutsches Zentrum Immuntherapie (DZI), Friedrich-Alexander-University Erlangen-Nürnberg (FAU) and Universitätsklinikum Erlangen, Erlangen, Germany

^3^Pattern Recognition Lab, Department of Computer Science, Friedrich-Alexander-Universität Erlangen-Nürnberg, Erlangen, Germany

^4^Department Artificial Intelligence in Biomedical Engineering, Friedrich-Alexander-Universität Erlangen-Nürnberg, Erlangen, Germany

^5^Department of Internal Medicine 3 – Rheumatology and Immunology, Friedrich-Alexander-University Erlangen-Nürnberg (FAU) and Universitätsklinikum Erlangen, Erlangen, Germany

^6^Department of Rheumatology and Clinical Immunology, Charité - Universitätsmedizin Berlin

**Supplementary Table 1.** Reader study. Pairwise Pearson r coefficients between readers with respective the confidence intervals and p values are shown.

|  |  | **Pearson r** | **CI95%** | **p value** |
| --- | --- | --- | --- | --- |
| Reader 1 | Reader 2 | 0.76 | [0.64, 0.85] | 5,65e-14* |
| Reader 1 | Reader 3 | 0.54 | [0.34, 0.69] | 2,54e-6* |
| Reader 1 | Reader 4 | 0.49 | [0.28, 0.65] | 2,24e-5* |
| Reader 1 | Reader 5 | 0.38 | [0.15, 0.57] | 1,49e-3* |
| Reader 2 | Reader 3 | 0.40 | [0.17, 0.58] | 8,92e-4* |
| Reader 2 | Reader 4 | 0.68 | [0.53, 0.79] | 1,23e-10* |
| Reader 2 | Reader 5 | 0.40 | [0.18, 0.58] | 7,94e-4* |
| Reader 3 | Reader 4 | 0.38 | [0.15, 0.56] | 1,57e-3* |
| Reader 3 | Reader 5 | 0.09 | [-0.16, 0.32] | 4,95e-1 |
| Reader 4 | Reader 5 | 0.32 | [0.08, 0.51] | 9,52e-3* |

**Supplementary Figure 1.** The modified Nail Psoriasis Severity Index (mNAPSI) evaluates psoriatic changes in the nail bed (onycholysis, oil spot discoloration, splinter hemorrhages, hyperkeratosis) and nail matrix (crumbling, pitting, leukonychia, red spots in the lunula) based on their presence and portion of nail involved. Clinical images as examples of these nail abnormalities are provided for nail bed changes (upper panel) and matrix changes (lowe panels).

**Supplementary Figure 2.** Side-by-side comparison of hand photographs acquired with the earlier standardized method using a light-reflection-free box (A1, A2) versus the current unstandardized acquisition method leading to uneven lighting and reflections (B1, B2)**.** Original hand photographs (A1, B1) and the extracted nail images (A2, B2) are shown.

**Supplementary Figure 3.** Graphical representation of inter-reader correlations between the 5 readers.
